# Effect of cannulation site on emboli travel during cardiac surgery

**DOI:** 10.1186/s13019-021-01564-1

**Published:** 2021-06-23

**Authors:** Mira Puthettu, Stijn Vandenberghe, Stefanos Demertzis

**Affiliations:** 1 Foundation for Cardiovascular Research and Education (FCRE), Cardiovascular Engineering, Istituto Cardiocentro Ticino, Via ai Söi 24, 6807 Torricella-Taverne, Switzerland; 2 Department of Cardiac Surgery, Istituto Cardiocentro Ticino, Lugano, Switzerland; 3 Faculty of Biomedical Sciences, Università della Svizzera Italiana, Lugano, Switzerland

**Keywords:** Air emboli, Cannulation site, Cardiac surgery, Bubble counter

## Abstract

**Background:**

During cardiac surgery, micro-air emboli regularly enter the blood stream and can cause cognitive impairment or stroke. It is not clearly understood whether the most threatening air emboli are generated by the heart-lung machine (HLM) or by the blood-air contact when opening the heart. We performed an in vitro study to assess, for the two sources, air emboli distribution in the arterial tree, especially in the brain region, during cardiac surgery with different cannulation sites.

**Methods:**

A model of the arterial tree was 3D printed and included in a hydraulic circuit, divided such that flow going to the brain was separated from the rest of the circuit. Air micro-emboli were injected either in the HLM (“ECC Bubbles”) or in the mock left ventricle (“Heart Bubbles”) to simulate the two sources. Emboli distribution was measured with an ultrasonic bubble counter. Five repetitions were performed for each combination of injection site and cannulation site, where air bubble counts and volumes were recorded. Air bubbles were separated in three categories based on size.

**Results:**

For both injection sites, it was possible to identify statistically significant differences between cannulation sites. For ECC Bubbles, axillary cannulation led to a higher amount of air bubbles in the brain with medium-sized bubbles. For Heart Bubbles, aortic cannulation showed a significantly bigger embolic load in the brain with large bubbles.

**Conclusions:**

These preliminary in vitro findings showed that air embolic load in the brain may be dependent on the cannulation site, which deserves further in vivo exploration.

## Background

Micro-emboli are widely reported to enter the cardiovascular system during cardiac surgery [1, 2]. These emboli, solid or gaseous, become particularly dangerous once they enter the cerebral circulation, leading to silent infarcts, stroke or, in the worst-case scenario, death [3–7]. In the study of Salazar et al., stroke is diagnosed in 3.6% of the patients who underwent various types of cardiac surgery [8]. The study of Anyanwu et al. proved that the incidence depends on the type of surgical procedure; in fact, stroke incidence decreases to 1.7% if only patients who underwent coronary artery bypass grafting (CABG) are considered [9]. One possible factor that leads to this reduction is the increased prevalence of air emboli in intracardiac procedures [10]. Many studies affirm also that gaseous micro-emboli (GME) might be one of the causes for neurocognitive deficits postcardiac surgery [3, 5]

Dr. Chung and colleagues were able to translate transcranial Doppler signals into a distribution of cerebral artery bubble activity and reported, for example, more than 6000 gas emboli being generated during a coronary surgery, with a bubble diameter ranging from 5 μm up to a few millimeters [1].

The origin of emboli during cardiac surgery is difficult to identify [11], but two possible sources of GME are the heart-lung machine [12] and the blood-air contact at the surgical site [13], where air bubbles form in the pulmonary veins, the heart or in the aorta, and then are pushed towards the periphery during the first heartbeats. Air bubbles can be created at different steps of the surgery [1, 14], but it is not clearly understood which is the most threatening source. An efficient method to prevent GME generation is still to be found. However, there are other factors that can be manipulated to reduce the amount of produced GME.

The goal of this in vitro study is to evaluate, whether the cannulation site for cardiopulmonary bypass (CPB) influences the GME load in the brain. Three frequently used cannulation sites (ascending aorta, axillary artery and femoral artery) are compared in terms of brain safety with a focus on air emboli.

## Methods

### Test setup

**Fig. 1:**
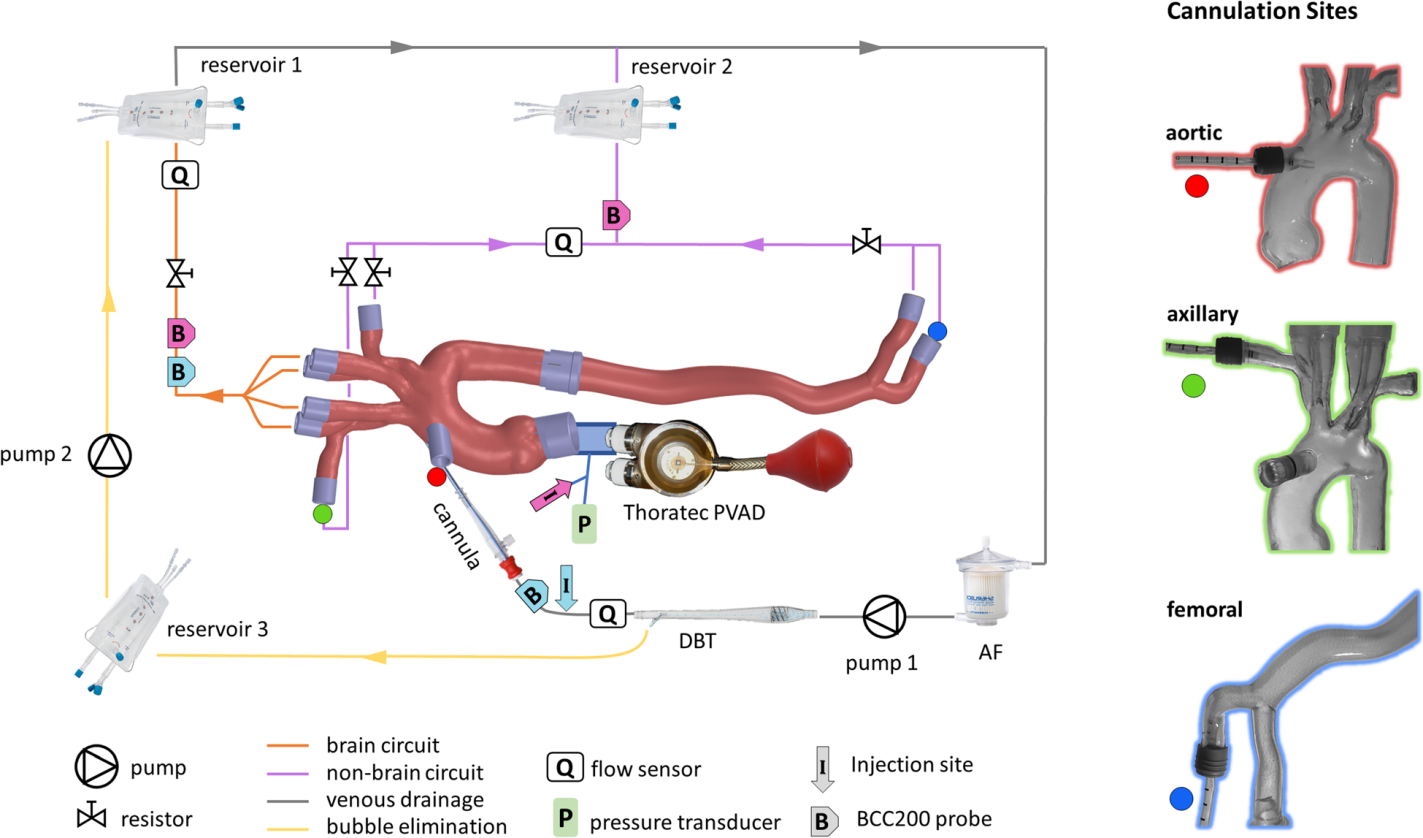
On the left: scheme of the circuit including 3D-printed model of the aorta and its main branches. It includes four lines: brain circuit (orange), non-brain circuit (purple), venous drainage (dark grey) and bubble elimination circuit (yellow). Insertion points for the three cannulation sites are shown as colored circles. In this scheme, aortic cannulation is shown. Location of injection site and BCC200 probes during ECC Bubbles generation and Heart Bubbles generation are shown in pink and in cyan, respectively. For each measurement, only one injection site and one cannulation site were used at a time. On the right: positioning of the 20Fr cannula during aortic cannulation (red), axillary cannulation (green), femoral cannulation (blue)

A hydraulic mockup of the human circulation during CPB was created (Fig. 1). The core was an aorta model created from CT scan and constructed with additive manufacturing (stereolithography) as 2 connecting parts from acrylic. The model contained the ascending aorta, aortic arch, descending aorta, and abdominal aorta. In addition, the onset of the supra-aortic vessels, including subclavian and vertebral arteries were presented, as well as the onset of the iliac arteries.

A Thoratec pulsatile ventricular assist device (labeled as “Thoratec PVAD” in Fig. 1) mimicked the left ventricle and was connected to the aortic root via a 1″ PVC tube (7 cm long), in which a pressure line (1 mm ID) was added as a side branch. A 85 mL rubber bulb was used to manually actuate the pneumatic Thoratec PVAD to simulate heart beats.

In CPB mode, flow of fluid into the aorta model was maintained by a turbodynamic pump (labeled as “pump 1” in Fig. 1; a Deltastream DP2, Medos Medizintechnik AG, Stolberg, Germany). It pumped the fluid through an arterial cannula (20Fr, Maquet Cardiopulmonary GmbH, Rastatt, Germany) that was inserted in one of the 3 cannulation sites in the aorta model via a sealed plug. Fluid exited the aorta model through 1/2″ and 3/8″ PVC tubing that connected the artery ends to one of 2 flexible reservoir bags (labeled as “reservoir 1” and “reservoir 2” in Fig. 1; V.V.R. 1800, Eurosets s.r.l., Medolla, Italy), via a series of merging connectors. As such, the circuit was eventually split in 2 main streams: fluid passing through vertebral and carotid arteries (“brain circuit”) going to reservoir 1, and subclavian and iliac arteries going to reservoir 2 (“non-brain circuit”).

Total flow rate coming from the pump was fixed at 4 L/min. Flow distribution in the model was regulated using ball valves on two parallel branches on the brain section and two Hoffman clamps for each subclavian branch. The target flow distribution was 15% in the brain, 25% in the arms and 60% in the descending aorta, based on the study by Mueller et al. [15]

A blood analog was used, consisting of 60% distilled water, 39.2% Glycerol (99.5%, APC Pure, Manchester, UK) and 0.8% of surfactant (Pluronic® F-68, AppliChem GmbH, Darmstadt, Germany). Its surface tension, determined by drop weight method, was 58.1 mN/m (±0.0028, *n* = 6). This method was previously validated using reference fluids, such as H_2_O and Glycerol. The dynamic viscosity was 3.1 cP (±0.025, *n* = 3) at 27.5 °C, and was evaluated using a U-tube viscometer (U-Tube Type BS/U size B/td>, Ubbelohde Capillary, Fungilab, Barcelona, Spain).

### Cannulation methods

The same 20 Fr arterial cannula was used for all three cannulation sites. For aortic cannulation (Fig. 1, top picture on the right), the tip of the cannula was pushed into the aortic arch via a specific port that was plugged, flush with the aortic lumen, for the other cannulation experiments. For axillary cannulation (Fig. 1, middle picture), the right arm’s tube was removed from the connector on the right subclavian artery and the cannula was placed instead. Its tip was placed just before the brachiocephalic trunk to prevent carotid obstruction. The same procedure was used for femoral cannulation in the right leg’s tube (Fig. 1, bottom picture). The tip of the cannula was pushed into the right leg before the iliac bifurcation.

### Air Bubbles Generation & Measurement

In order to generate air bubbles with a similar size distribution to air emboli found in patients during cardiac surgery, the following method was used.

A 10 mL syringe containing 3 mL of blood analog was connected to a 5 mL syringe containing 1 mL of air, via a 3-way valve. The content of the two syringes was shuttled back and forth for a total of 20 times so the air was dispersed mostly as microbubbles in the final fluid. The syringe with the mixture was then rested vertically with the tip pointing downward for 4 min, in order to allow foam and big bubbles to collect on the top part. Next, 0.5 mL were disposed of and 1 mL was then immediately injected into the circuit, leaving the remaining mixture and the top foam in the syringe.

Two sites of injection were used during experiments: i) upstream of the cannula to simulate air bubbles coming from the heart-lung machine (“ECC Bubbles”), and ii) in the side port of the pressure line before the Thoratec pump to simulate air bubbles that collect in the heart and get ejected in the first beat (“Heart Bubbles”). This second injection type was coordinated with compression of the rubber bulb, in order to simulate the first heartbeat after reperfusion.

In order to compare cannulation sites, bubble counts and corresponding volumes were measured by a Bubble Counter (BCC200, GAMPT mbH, Merseburg, Germany) with two probes for standard 3/8″ PVC tubing. One probe was always placed on the brain collecting branch to evaluate cerebral gas emboli. When evaluating ECC Bubbles, the other probe was positioned immediately downstream of the injection site to quantify total injected air bubbles. During Heart Bubbles evaluation, where the injection site is a 1″ tube, this second probe was moved to the non-brain region of the model to measure gas emboli in arms and legs.

Following an injection of bubble mixture, a peak was visible in the bubble count plot on the BCC200 screen as bubbles pass through the probes. For each cannulation site, a total of ten acceptable peaks were recorded and analyzed: five during ECC Bubbles generation and five during Heart Bubbles generation. A peak was considered acceptable, when reaching at least 60 bubbles/s during ECC Bubbles injection and at least 3 times the initial baseline count during Heart Bubbles injection. All recordings with a baseline count higher than 20 bubbles/s before injection or containing more than 10 overrange bubbles (diameter > 500 μm) were excluded and redone.

For reducing the baseline count, an arterial filter (labeled as “AF” in Fig. 1; Sherlock mini, Eurosets s.r.l., Medolla, Italy) was placed upstream of pump1 to reduce presence of big bubbles. Additionally, a Dynamic Bubble Trap (labeled as “DBT” in Fig. 1, Kardialgut GmbH, Axtbrunn/Petersdorf, Germany) was installed downstream of pump 1 and upstream of the cannula. The suction line of the DBT is normally connected to the venous reservoir during surgery. In our model, an additional circuit (“reservoir 3” and “pump 2” in Fig. 1) was built to re-pump the blood analog into the main circuit (“reservoir 1”), while the bubbles were eliminated at the reservoir surface.

### Instrumentation and other measurements

A single pressure transducer (TruWave, Edwards Lifesciences, Nyon, Switzerland) was used to measure arterial pressure in the circuit and placed at the aortic root, close to the injection site for Heart Bubbles.

Three different transit-time ultrasonic flow sensors were used to measure total flow coming from the cannula, generated by pump 1, and flow rates in the brain and in the arms. The first two sensors (9XL, Transonic Systems Inc., NY, USA) were set upstream of the cannula and on the main brain tube. The last one (EM Tec, Finning, Germany) was put on the collecting tube of the arms branches. Flow rate in the descending aorta was calculated from the other measurements.

The analog outputs from all devices were digitized by an iWorx 416 data acquisition device (iWorx Inc., Dover, NH) and recorded on a computer with Labscribe 2 software (iWorx) at 200 Hz.

Before each experimental run, the pressure transducer was calibrated with a TruCal™ Tester (Edwards Lifesciences, Irvine, CA). All flow meters were gravimetrically calibrated (“bucket and stopwatch” method) once before the study with the help of a progressive cavity pump (MAE25, CSF Inox spa, Milan, Italy).

### Processing & Statistics

A first analysis of the peaks was done directly in the BCC200, where a time interval around the injection peak was selected from the whole recording and the corresponding bubble size histograms were saved.

All data collected from the BCC200 were processed and analyzed in Matlab 2019a (The MathWorks Inc., Natick, MA). The histograms were restructured in smaller bins: small bubbles (S, 10–40 μm), medium bubbles (M, 41–100 μm) and large bubbles (L, 101–500 μm). Afterwards, new counts and volumes of air bubbles were calculated for each peak, summing up all values of the selected range of bubble diameters.

At the end of the processing phase, for each category of bubbles (S, M and L), four parameters were computed for each combination of injection site and cannulation site. In the case of ECC Bubbles: total bubble count, total bubble volume, brain bubble count and brain bubble volume. Meanwhile for Heart Bubbles: brain bubble count, brain bubble volume, non-brain bubble count and non-brain bubble volume. Since a large inter-variability was observed between bubble injections, bubble counts and volumes were normalized to the corresponding total bubble volume injected. Bubble counts were then expressed as bubble densities (bubbles/μL) and bubble volumes as ratios of the total injected volume (μL/μL).

Statistical tests were carried out to compare cannulation sites based on the bubble counts and volumes, both normalized to the injected bubble volume.

A paired t-test was used (significance level *p* < 0.05) if normality was confirmed with a Kolmogorov-Smirnov test. For non-normal distributions, a Wilcoxon-Mann-Whitney test was performed for each pair compared.

Additionally, mean pressure and mean flow rates from cannula, brain and arms of the whole recording were calculated for each cannulation site.

## Results

One important observation relates to the impact of cannulation site on the flow distribution in the model, reported in Table 1.

**Table 1:** Summary of arterial pressure and flow distribution for the three cannulation sites. Total flow (Q_Total_) was measured at the ECC cannula, and the local flow rates were also reported as a percentage of this flow rate. Q_Brain_: combined flow rate through carotid and vertebral arteries; Q_Arms_: combined flow rate through left and right arms; Q_Legs_: combined flow rate from abdominal artery into left and right legs

	Pressure	Q_**Total**_	Q_**Brain**_		Q_**Arms**_		Q_**Legs**_	
Cannulation	[mmHg]	[L/min]	[L/min]	[%]	[L/min]	[%]	[L/min]	[%]
Aortic	38.9 ± 1.9	4.01 ± 0.08	0.66 ± 0.04	16.5	0.94 ± 0.04	23.4	2.39 ± 0.09	59.6
Axillary	37.1 ± 3.4	4.01 ± 0.08	0.61 ± 0.06	15.2	0.22 ± 0.01	5.5	3.17 ± 0.10	79.1
Femoral	37.0 ± 2.3	4.05 ± 0.08	0.64 ± 0.04	15.8	1.10 ± 0.05	27.2	2.30 ± 0.08	56.8

The target flow distribution, achieved during aortic cannulation, was: ~ 15% of the total flow to the brain, ~ 25% to the arms and then the rest, ~ 60%, to the descending aorta. Peripheral resistances were not changed after this initial setting, but an alteration in the flow distribution was observed in both other cannulation sites.

During axillary cannulation, where the left arm was blocked by the cannula, there was a drastic increase of flow in the legs. Arms’ flow rate decreased to a mean percentage of 5.5%, while legs’ flow increased to a mean percentage of 79.1%.

During femoral cannulation, in contrast, the changes were less drastic even though the left leg was blocked and a retrograde flow was observed in the descending aorta. The arms’ flow increased up to a final mean value of 27.2%, while the mean flow in the right leg was 56.8%, which yielded to a flow distribution close to our target values.

Since flow distribution was not constant among different cannulation sites, correlation coefficients were calculated between percentage of total flow rate and corresponding measured air bubble volume among all measurements, both for brain and non-brain (arms+legs) regions. Correlation coefficients were obtained for the three ranges of bubbles: small (S), medium (M) and large (L).

The results showed that there was no correlation for S and M bubbles. A small positive correlation was found for L bubbles, both in the brain region (r = 0.4317) and in the non-brain region (r = 0.4498).

**Fig. 2:**
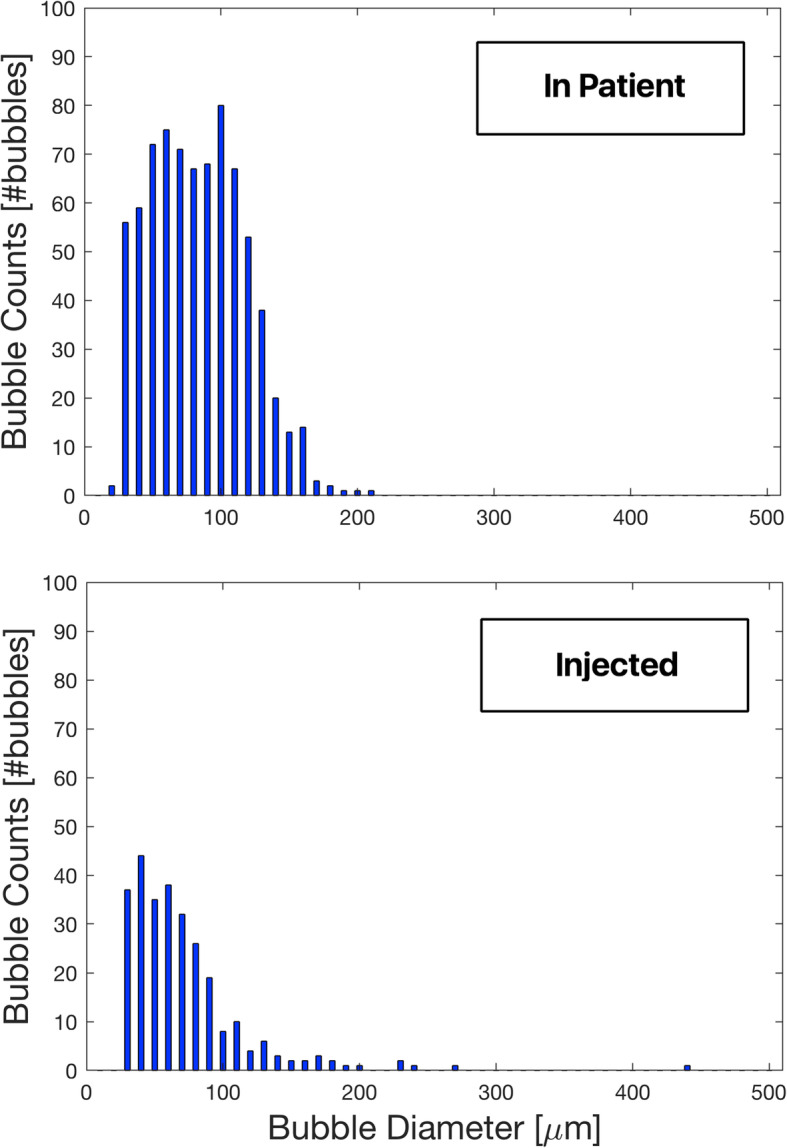
Bubbles size distribution in a surgical case (top) and in one injection in the in vitro model (bottom). Bins are 10 μm wide

Figure 2 shows size distribution of air bubbles injected during this study, compared to a bubble histogram from an actual patient case (demo file provided by Gampt GmbH).

In both cases, histograms were obtained from selecting an isolated peak in the bubble count vs. time graph, which in this study represented injection of air bubbles and in the case of patient represented surgical manipulation.

The two histograms are comparable in terms of S bubbles, but bubble distribution measured in the patient shows a wider spread to the right, indicating more M and L bubbles. Injected air bubbles contained few L bubbles and the majority were S and M bubbles with a peak at 50 μm.

Bubbles smaller than 10 μm in diameter are not shown due to the sensitivity of the bubble counter.

Five bubbles injections were performed for each combination of injection site and cannulation site, with a total of 30 injections. Mean values and standard deviation of bubble counts and volumes normalized to the total injected bubble volume per injection are shown in Table 2. For Heart Bubbles, the total injected bubble count is not available. Bubbles can break down or merge during circulation and thus number preservation between upstream injection and downstream measurement cannot be assumed. However, in the assumption that no bubbles got trapped in the model, a preservation of bubble volume enables to derive the total injected bubble volume, which is therefore chosen for the normalization of the data.

**Table 2:** Mean values and corresponding standard deviation for all measurements of bubble counts (bubbles/μL) and bubble volumes, expressed as percentages for readability (μL/μL*100). Values are shown for total injected bubbles, brain branch and non-brain region (arms + legs), and are normalized by the total injected volumes of bubbles. During ECC Bubbles injection, information on non-brain counts was not available, meanwhile during Heart Bubbles injection, total injected counts were not recorded. The same is valid for volumes values, but missing information was calculated due to conservation of mass

**BUBBLE COUNT [bubbles/μL]**								
Injection Site	ECC Bubbles				Heart Bubbles			
Bubbles Size	**S**	**M**	**L**	**SUM**	**S**	**M**	**L**	**SUM**
Aortic Cannulation								
Injected	0.38 ± 0.31	0.54 ± 0.47	0.23 ± 0.11	1.15	N/A	N/A	N/A	N/A
Brain	0.12 ± 0.07	0.16 ± 0.09	0.1 ± 0.07	0.38	0.41 ± 0.25	0.43 ± 0.17	0.16 ± 0.02	1
Non-Brain	N/A	N/A	N/A	N/A	0.83 ± 0.58	0.63 ± 0.13	0.24 ± 0.03	1.7
Axillary Cannulation								
Injected	0.27 ± 0.08	0.42 ± 0.17	0.23 ± 0.08	0.92	N/A	N/A	N/A	N/A
Brain	0.15 ± 0.06	0.23 ± 0.09	0.06 ± 0.02	0.39	0.2 ± 0.24	0.23 ± 0.27	0.05 ± 0.03	0.48
Non-Brain	N/A	N/A	N/A	N/A	0.63 ± 0.69	0.43 ± 0.39	0.13 ± 0.03	1.19
Femoral Cannulation								
Injected	0.09 ± 0.05	0.15 ± 0.04	0.14 ± 0.03	0.38	N/A	N/A	N/A	N/A
Brain	0.03 ± 0.01	0.08 ± 0.01	0.05 ± 0.01	0.16	0.84 ± 1.36	0.84 ± 1.42	0.08 ± 0.04	1.76
Non-Brain	N/A	N/A	N/A	N/A	2.58 ± 4.63	1.14 ± 1.34	0.24 ± 0.13	3.96
BUBBLE VOLUME DISTRIBUTION [μL/μL*100]								
Injection Site	ECC Bubbles				Heart Bubbles			
Bubbles Size	**S**	**M**	**L**	**SUM**	**S**	**M**	**L**	**SUM**
Aortic Cannulation								
Injected	0.6 ± 0.47	8.36 ± 7.29	91.0 ± 7.7	100%	1.86 ± 1.26	17.65 ± 3.24	80.49 ± 3.79	100%
Brain	0.18 ± 0.10	2.62 ± 1.63	40.62 ± 53.57	43.42%	0.66 ± 0.44	7.01 ± 2.32	42.40 ± 8.42	50.07%
Non-Brain	0.42 ± 0.39	5.74 ± 6.15	50.42 ± 59.29	56.58%	1.20 ± 0.84	10.63 ± 2.74	38.09 ± 8.82	49.92%
Axillary Cannulation								
Injected	0.42 ± 0.14	6.4 ± 2.2	93.18 ± 2.34	100%	1.27 ± 1.37	8.4 ± 7.62	90.34 ± 8.96	100%
Brain	0.23 ± 0.09	3.63 ± 1.33	14.89 ± 8.59	18.75%	0.32 ± 0.37	3.32 ± 3.77	14.97 ± 6.62	18.61%
Non-Brain	0.19 ± 0.09	2.77 ± 2.85	78.29 ± 8.05	81.25%	0.95 ± 1.0	5.08 ± 3.95	75.37 ± 13.32	81.4%
Femoral Cannulation								
Injected	0.13 ± 0.06	2.56 ± 0.47	97.31 ± 0.51	100%	4.94 ± 8.73	24.83 ± 25.78	70.22 ± 34.3	99.9%
Brain	0.04 ± 0.01	1.43 ± 0.2	15.56 ± 4.95	17.03%	1.25 ± 2.12	10.65 ± 15.92	16.73 ± 12.98	28.63%
Non-Brain	0.08 ± 0.05	1.13 ± 0.39	81.76 ± 5.21	82.97%	3.69 ± 6.61	14.18 ± 10.38	53.49 ± 31.23	71.36%

It can be observed that the origin of the bubbles (injection site) played a role in the size distribution of bubbles in terms of volumes. For ECC Bubbles overall, 94% of the injected bubble volume came from large bubbles (L), 5% from medium-sized ones (M) and only 1% from small ones (S). Meanwhile, during Heart Bubbles injection, large bubbles accounted for 80% of the total injected volume, medium size ones for 17% and small bubbles for 3%. However, in terms of counts normalized to total volume, majority of bubbles appeared to be small or medium. For the brain region, counts of Heart Bubbles were higher than counts of ECC Bubbles, which was also reflected in the bubble volumes. This difference can be a consequence of the applied technique for bubble generation.

Kolmogorov-Smirnov tests confirmed normal distribution of bubble counts and volumes for all cannulation sites and all types of injection (α = 0.01). For this reason, paired t tests were used to compare cannulation sites based on M and L bubbles. S bubbles were neglected since are considered non-dangerous for the brain, according to literature [1].

For ECC Bubbles, maximum values for bubble count and volume in the brain were recorded during axillary cannulation for M bubbles (bubble count = 0.23 bubbles/μL, bubble volume = 3.6%) and during aortic cannulation for L bubbles (bubble count = 0.1 bubbles/μL, bubble volume = 40.6%). Minimum bubble count and volume in the brain were observed during femoral cannulation for M bubbles (bubble count = 0.08 bubbles/μL, bubble volume = 1.4%). L bubbles in the brain were fewest during femoral cannulation (bubble count = 0.05 bubbles/μL), but the lowest bubble volume was observed during axillary cannulation (bubble volume = 14.9%). Paired t-tests confirmed that femoral is significantly better than axillary cannulation for medium ECC Bubbles (*p* < 0.01). No significant difference was observed for L bubbles. Aortic cannulation was comparable to the other two locations for both bubble sizes.

For Heart Bubbles, maximum values for bubble count and volume in the brain were recorded during femoral cannulation for M bubbles (bubble count = 0.84 bubbles/μL, bubble volume = 10.6%) and during aortic cannulation for L bubbles (bubble count = 0.16 bubbles/μL, bubble volume = 42.4%). Minimum values of bubble count and volume in the brain were observed during axillary cannulation for M bubbles (bubble count = 0.23 bubbles/μL, bubble volume = 3.3%). For L bubbles, the lowest bubble count and volume in the brain were recorded during axillary cannulation (bubble count = 0.05 bubbles/μL, bubble volume = 15.0%). Paired t-tests confirmed that aortic cannulation resulted in a significantly bigger amount of large Heart Bubbles than axillary and femoral cannulations (*p* < 0.01), but no significant difference was found for M bubbles.

**Fig. 3:**
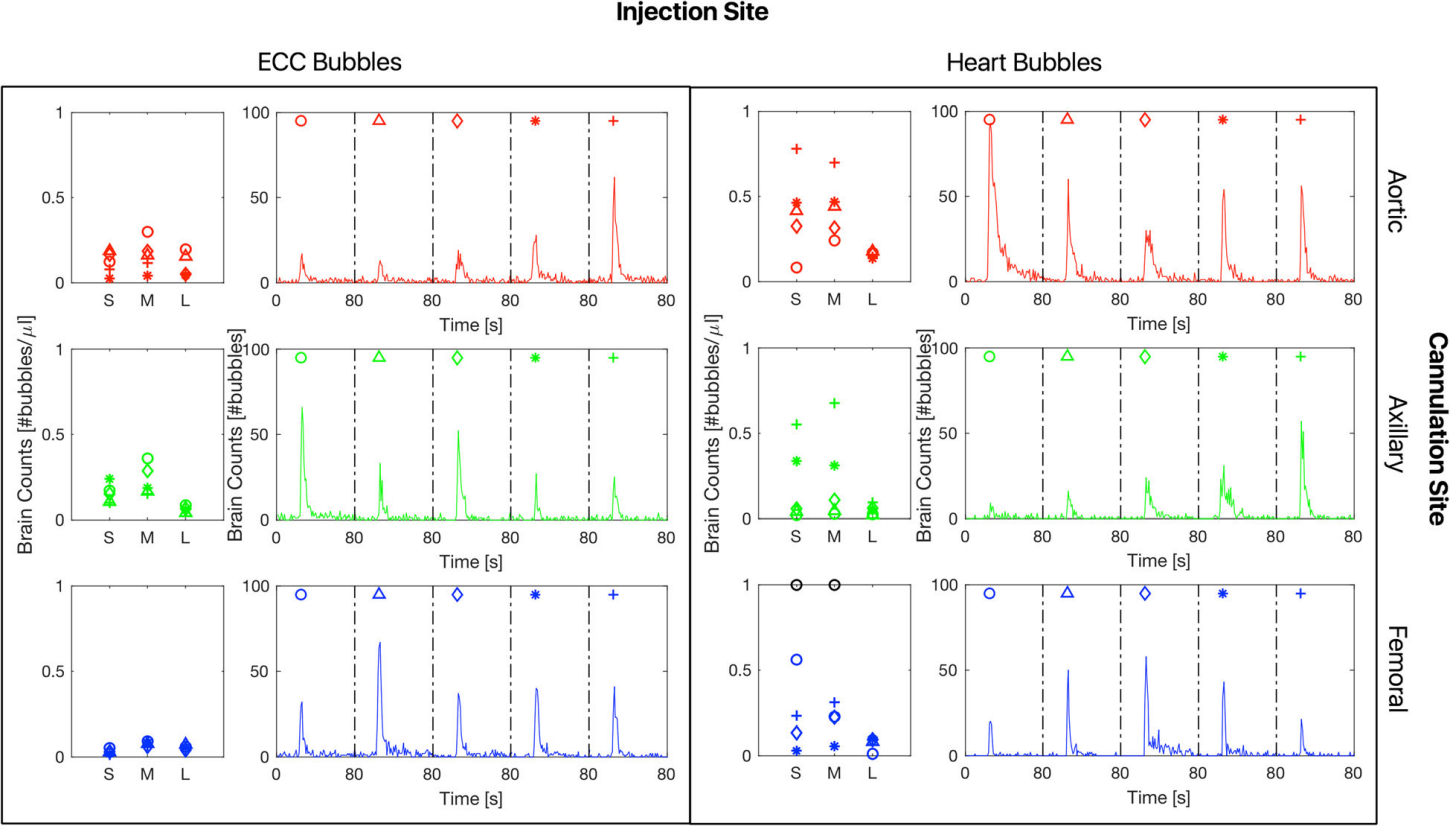
For each combination of injection site and cannulation site, two plots are shown. On the left: brain bubble counts normalized to total injected bubble volume, expressed in bubbles/μL. Bubble counts were separated for small bubbles (S), medium bubbles (M) and large bubbles (L). Each symbol represents a single bubble injection (5 repetitions in total for each pair). Outliers are shown with black circles (Heart Bubbles; femoral cannulation; for S bubbles: 3.26 bubbles/μL, for M bubbles: 3.38 bubbles/μL). On the right: brain bubble counts (not normalized) during recorded peaks for each repetition (corresponding symbol is put on top). All peaks are shown over a time interval of 80 s

An overview of bubble counts that reached the brain is presented in Fig. 3, showing a comparison between ECC Bubbles and Heart Bubbles for all three cannulation sites. Each symbol represents a single bubble injection.

In general, there was a lot of variance between injections and for all bubble sizes, especially for Heart Bubbles. This can be seen also from the fact that a big peak in absolute bubble count did not lead necessarily to a big bubble density (counts normalized by total injected volume).

When focusing on normalized bubble counts (bubbles/μL), femoral cannulation showed the smallest variance for ECC Bubbles (all sizes, standard deviation = 0.01 bubbles/μL), while conversely it showed the largest variance for Heart Bubbles (M, standard deviation = 1.42 bubbles/μL).

Comparison between cannulation sites in terms of cerebroprotection based on Fig. 3 is consistent with Table 2.

**Fig. 4:**
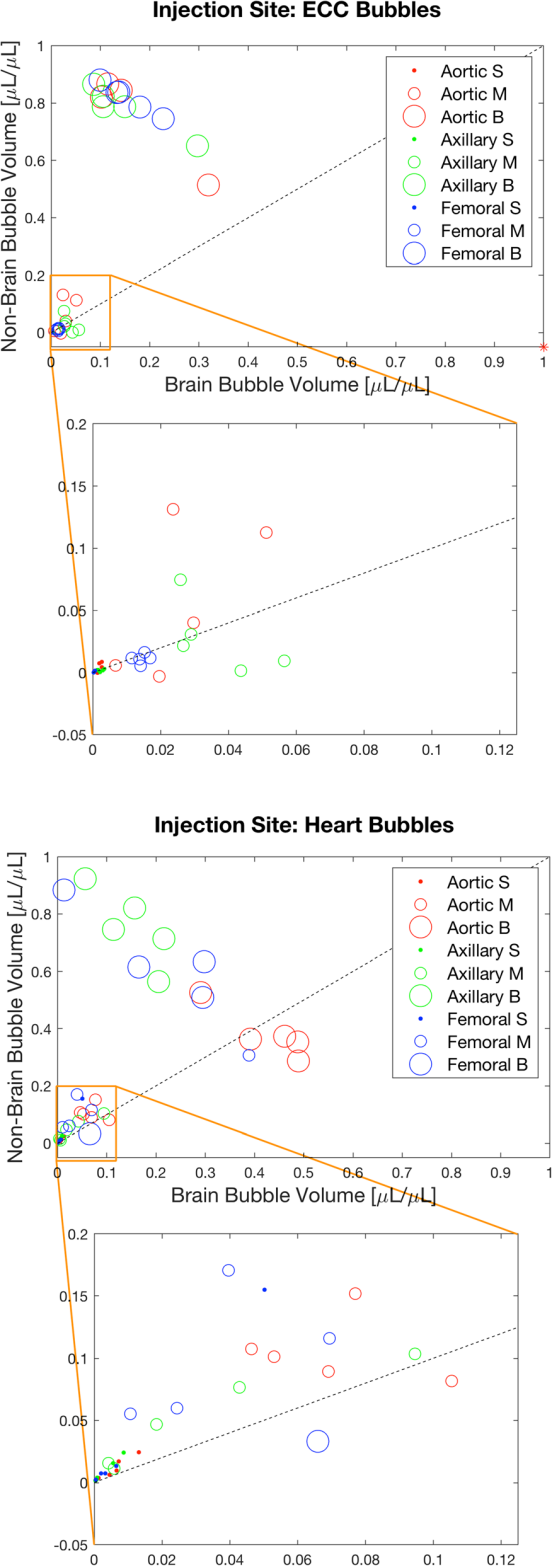
Brain bubble volumes and non-brain bubble volumes were normalized to total injected bubble volumes, and expressed as ratios (μL/μL). Results are shown for all cannulation sites and all size categories of bubbles (small, S; medium, M; and large, L). Dotted black line represents a perfect split between brain region and non-brain region, which means 50% went to one part and the rest to the other one. A zoom for small and medium bubbles is also shown. Top: bubbles injection in the HLM circuit (ECC Bubbles), bottom: bubbles injection in front of heart model (Heart Bubbles). The outlier is shown with a red star (ECC generated L Bubbles; aortic cannulation; Brain bubble volume = 135.15 μL/μL, Non-brain bubble volume = − 52.48 μL/μL)

An overview of normalized bubbles volumes is shown in Fig. 4. For each single bubble injection, three data points were plotted (one for each size category: S, M and L).

Ratio of total bubble volume that went to the non-brain region was plotted against ratio of total bubble volume that went to the brain region; the dotted line represents an even distribution between the two areas of the model (50% to the brain, 50% to the non-brain).

In general, L bubbles are located mainly on the left side of the dotted line, which means that they preferred to go in the non-brain region, independently of the cannulation site. This observation is not true for aortic cannulation during Heart Bubbles injection, where 4 injections over 5 are on the right side of the dotted line. This preference is shown also for S and M bubbles during Heart Bubbles injection, but they were more distributed between brain and non-brain regions during ECC Bubbles injection.

Nevertheless, even though the biggest amount of bubble volume went to the non-brain region, it does not implicate that none went to the brain.

## Discussion

According to Chung and colleagues [1], a significant amount of air bubbles, ranging from a few hundreds to a few thousands, enters the bloodstream and reaches cerebral areas during cardiac surgery. Air emboli recorded by trans-cranial Doppler ultrasound showed a wide size spectrum, with a majority of bubbles smaller than 100 μm in diameter. According to their predictions on dissolution times, air bubbles smaller than 38 μm would disappear before reaching the brain, and form no cerebral hypoxia risk, in contrast to bubbles > 38 μm.

The purpose of this in vitro study was to assess the effect of cannulation site on air emboli travel during cardiac surgery, and to compare three different cannulation sites. In order to determine the safest one from a cerebroprotection perspective, cannulation sites were statistically compared based on bubble count and volume measured in cerebral supply vessels. For this reason, S bubbles (10–40 μm) recorded in this study are not relevant for our purpose and will not be considered in the following discussion.

Based on the obtained results, a significant difference was found among cannulation sites for both air bubbles sources. For each injection site, a different cannulation site appeared to provide the best brain safety and led to the least amount of air to the brain. For ECC Bubbles, where injection was done in the HLM circuit, the lowest amount of dangerous air bubbles (M bubbles) in the brain region was observed during femoral cannulation; however, aortic cannulation was statistically comparable. Axillary cannulation led to a significantly larger air embolic load in the brain. L bubbles are expected to be rare from the ECC in a clinical case, especially with continuous improvement in ECC components, so results found for M bubbles deserve most attention.

For Heart Bubbles, where injection was done in the proximity of the mock left ventricle, aortic cannulation resulted in the biggest amount of dangerous air bubbles (L bubbles) in the brain. Axillary and femoral cannulations were statistically comparable in terms of cerebroprotection.

Other studies confirmed, consistently with our results, a dependence between observed embolic load (solid and gaseous) in the brain and the cannulation site. According to Hedayati et al., based on their in vivo study with microspheres performed in mongrel dogs, axillary cannulation is cerebroprotective during median sternotomy and isolation of the right axillary artery, due to a possible retrograde brachiocephalic artery blood flow produced by alterations in blood-flow patterns. Emboli distribution was determined a posteriori from tissue samples of middle cerebral arteries [16]. In the clinical study by Lei et al., they performed a multivariate logistic analysis on collected data from patients, including several physiological parameters pre- and during surgery, and found that femoral and axillary cannulations were comparable during type A aortic dissection surgery [17]. No significant difference between these two cannulation sites was found in the retrospective observational study by Kerdany et al. [18], either. The selected patients underwent redo valve surgery or denovo thoracic aorta surgery. In our study, this was true only during Heart Bubbles injection (i.e. during open heart surgery) but not for ECC Bubbles, where axillary cannulation resulted in the biggest air embolic load in the brain.

In a clinical case, differences between cannulation sites are dependent also on other factors that may affect the results obtained, such as cannula size, orientation, depth and flow rates.

Most of these factors were limited as much as possible in our study. The same cannula size was used for all cannulation sites. Moreover, once inserted in the model, the cannula was not moved during injections for the same cannulation site. So, it is interesting to notice that the effect of cannulation site works in different ways for the differently generated bubbles in our model: for ECC Bubbles, a different cannulation site means a different location where bubbles were released in the circulation. For Heart Bubbles, a different cannulation site means that even though the bubbles are always released at the same location, the altered streamlines in the circulatory system distribute the bubbles differently for each cannulation position.

Concerning the impact of flow rate on the results, it was not possible to have an absolutely identical flow distribution for all cannulation sites, because vasomotor control was not simulated and afterload resistances were kept constant, while the different cannulations obstructed different vessels. However, no detailed clinical information on resistance changes and flow rate distribution during CPB is available, so a similar behavior may be presented clinically [19, 20].

On the other hand, percentage of flow rate going to the brain was quite constant among different cannulation sites and correlation coefficients between flow rate and corresponding measured bubble volume, both for brain and non-brain, showed a small positive value uniquely for L bubbles (101–500 μm). This last finding is consistent with previous studies that demonstrated a strong size-destination relationship only for bubbles bigger than 1 mm in diameter [21].

Additionally, according to our results, dangerous bubbles generally preferred to go in the non-brain region, independently of the cannulation site, with the only exception for aortic cannulation during Heart Bubbles injection, which happens to be the most common cannulation technique for open heart surgery.

The conclusions that can be drawn from this in vitro study are consistent with previous findings. Nevertheless, there are certain limitations that should be considered.

Besides the above-mentioned inconsistent flow distribution for the different cannulation sites, other limitations of the 3D printed model include the lack of compliance, which would only impact pulsatile flow, and the lack of vessel collapse. The model is also limited to the main arterial branches, where the biggest simplification is that all flow downstream of the aortic arch goes to the legs. This enabled us to measure all bubbles in the circuit and separate brain vs. non-brain bubbles, where we assumed that all non-brain bubbles are “safe”. In reality, the extensive arterial non-brain network means that bubbles could enter distal organs where tissue damage or inflammatory reactions are also possible.

Some differences were observed between ECC Bubbles, which simulated air emboli originated in the HLM, and Heart Bubbles coming directly from the heart, and then enter the system during surgical manipulations. In general, Heart Bubbles had a bigger variance among injections and for all bubble sizes, except for L bubbles in the brain. Additionally, they resulted in a higher bubble count and volume in the brain region than ECC Bubbles.

A difference in size distribution was also observed between ECC Bubbles and Heart Bubbles, where in the former large bubbles accounted for 94% of the total injected volume and in the latter only for 80%. The above differences can be attributed to the injection method for Heart Bubbles having one additional step (generating ejection of the PVAD pump) and the fact that bubbles could not be measured directly at the injection site.

On the other hand, a larger variation is expected between patients in the clinical case, where many additional factors come to play, such as equipment, procedure and others.

Another hypothesis can explain the difference in large bubble content. Preliminary tests where larger bubbles acted as visible tracers demonstrated a clear turbulent region in the ascending aorta, which is also present physiologically [22] and may lead to the breakdown of large bubbles into smaller ones. For this reason, the hypothesis that total injected bubble counts were the sum of brain bubble counts and non-brain bubble counts was rejected and data analysis was based on the assumption of volume preservation.

Moreover, for aorta and axillary cannulations, some bubbles were observed to be trapped in a whirl and spun around the aorta circumference for several seconds, giving them more time to break down or dissolve. This brings about another parameter that we could not study at this time: the residence time of air emboli in large vessels. This is likely strongly related to the orientation of the cannula and a longer residence time may give emboli more time to partially dissolve and shrink to a safer size.

The comparison between bubbles injected in this study and air bubbles found in a typical patient showed that there may be more M and L bubbles in this last case. This indicates that our injected bubble mixture was relatively “safe” compared to the bubble distribution actually present in this particular real patient case. The distribution of air bubbles injected in our model also showed large variability and thus reduced our power to detect significant differences. This variability can be explained by the complicated method to produce mixtures with air micro-emboli, but a wide variation in generated bubble counts and sizes is also to be expected during surgery where many factors play a role.

The bubble size distribution found by Chung and colleagues based on all emboli signals recorded in their study during cardiopulmonary bypass, which includes both CABG surgery and valve replacement surgery, was similar to ours during ECC Bubbles injection (where data about total injected bubble count was available). In both cases, majority of recorded bubbles were smaller than 100 μm and M bubbles were frequent. These observations are not consistent with the findings of Schonburg et al., where majority of recorded bubbles were smaller than 40 μm in diameter, with a peak at 20 μm, for both analyzed patients [14].

We suspect that, due to improving filter capacity of CPB components or to breaking down of large bubbles [23], ECC Bubbles (even though they are clearly present) are generally less dangerous than Heart Bubbles because of their presumed smaller size. Nevertheless, the possibility that dangerous air bubbles are generated during cardiac surgery cannot be excluded a priori and necessary precautions should be taken.

The blood analog fluid used in this study was matched to viscosity, density and surface tension of normal blood. During CPB, dilution and cooling of the blood will affect these physical properties and may alter the processes of bubble break up, clustering and transport. We believe that the added complexity of simulating these changing physical properties in the fluid does not justify the expected minor differences in the results, but this should be verified in separate tests.

## Conclusions

Based on the here presented results, the chosen cannulation site in our in vitro model affected the portion of air bubbles traveling to the brain. This observation leads to the hypothesis that, by choosing an optimal cannulation site, air embolic load in the brain may be reduced also in clinical cases. This finding can be a starting point to guide future studies. Our findings represent unique preliminary results with accurate instruments that cannot be directly used on patients’ blood vessels. The model is a simplification of reality and these results should be verified in in vivo studies, with the best alternative measuring methods available such as trans-cranial Doppler ultrasound or brain imaging.

## Data Availability

-.

## Abbreviations

HLM: Heart-lung machine
ECC: Extra-corporeal circulation
CABG: Coronary artery bypass grafting
GME: Gaseous micro-emboli
CPB: Cardiopulmonary bypass
